# Thrombosis of mechanical mitral valve prosthesis during pregnancy: An ongoing “saga” in need of comprehensive solutions

**DOI:** 10.21542/gcsp.2020.32

**Published:** 2020-12-31

**Authors:** Ahmed Mahgoub, Susy Kotit, Karim Bakry, Ahmed Magdy, Hatem Hosny, Magdi Yacoub

**Affiliations:** 1 Aswan Heart Centre (AHC), Aswan, Egypt

## Abstract

Emergency treatment for thrombosed mechanical valve prothesis during pregnancy is not uncommon in low- and middle-income countries. The presence of a mechanical valve continues to be an important cause of maternal morbidity and mortality. There is a pressing need for increasing awareness and feasible solutions for this huge problem. We here describe four patients who needed emergency treatment for thrombosis of mechanical valve prothesis during pregnancy and review the evolving comprehensive strategies for dealing with this issue.

## Introduction

The presence of a mechanical heart valve during pregnancy poses one of the greatest clinical challenges due to the increased risk of valve thrombosis and the impact of anticoagulation on maternal and fetal morbidity and mortality^1–5^.

Maternal cardiovascular risk is increased in the presence of a mechanical valve (WHO risk classification III)^2,6–8^, and anticoagulation regimens carry an increased risk of miscarriage, hemorrhagic complications, and teratogenicity^9,10^. The chances of an event-free pregnancy with a live birth in the presence of a mechanical prothesis is only 58%^3^ and favorable outcome for mother and baby is only seen in 28% of the cases^11^.

We here describe four patients who required emergency treatment for thrombosed mitral valve prothesis, discuss complications related to mechanical valve prothesis during and after pregnancy and enumerate possible comprehensive solutions.

## Patients and Methods

Four patients presented in cardiogenic shock requiring emergency treatment for thrombosis of the mechanical valve prothesis during pregnancy.

### Patient 1

A 34-year-old female was referred due to thrombosis of a mechanical mitral valve prosthesis. There was history of five normal deliveries prior to mitral valve replacement, with no significant events. After the prosthetic valve, she had unexpected pregnancy twice, despite using different contraception methods.

The first pregnancy was terminated at 28 weeks due to severe vaginal bleeding and in the 14^th^ week of the second pregnancy she was admitted to obstetric unit for vaginal bleeding and threatened abortion, and was shifted to unfractionated heparin. On the fourth day of admission, vaginal bleeding increased and the unfractionated heparin was stopped. Within a few days, the mechanical valve thrombosed, and she developed progressive dyspnea, followed by cardiogenic shock, requiring non-invasive CPAP and vasopressor therapy.

**Figure 1.**
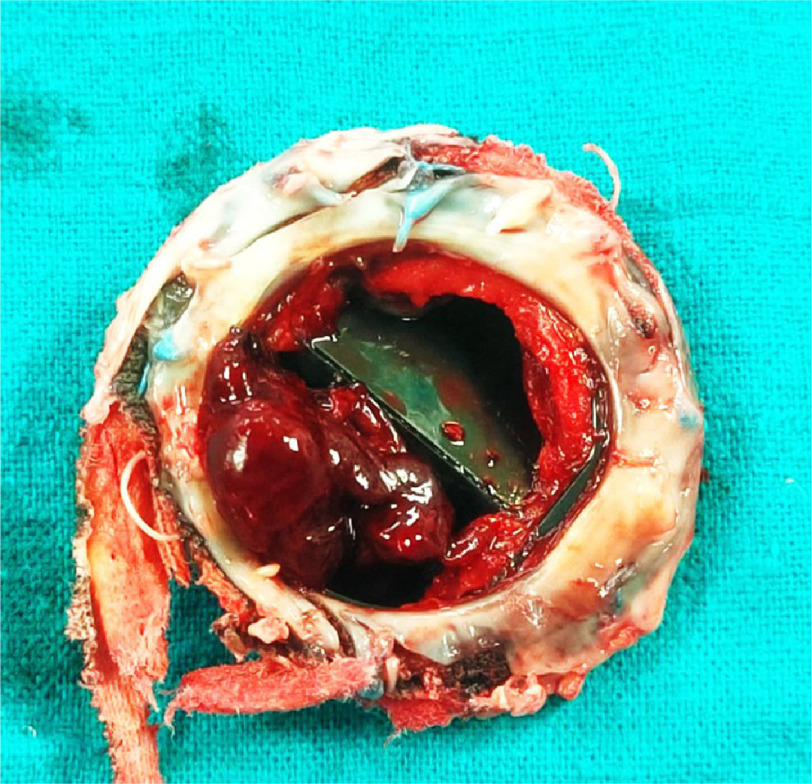
Mitral valve prosthesis showing large thrombus (atrial surface) in addition to large pannus.

**Figure 2.**
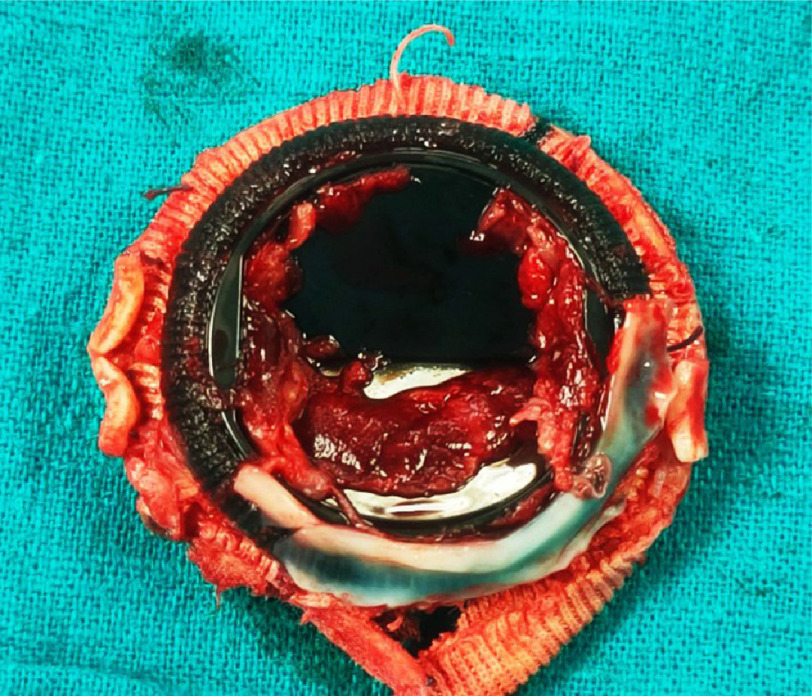
Mitral valve prosthesis showing large thrombus (ventricular surface).

The patient was transferred to the Aswan Heart Centre (AHC) and went to the OR immediately. Intra-operative trans-esophageal echocardiography showed malfunctioning mitral valve prosthesis with restricted mobility of both leaflets. Excision of the thrombosed mitral prosthesis showed thrombus on both atrial (Figure 1) and ventricular sides (Figure 2) of the prosthesis. A replacement operation was successfully performed with a mechanical St. Jude 31 mm showing with normal flow across the valve post-operatively. Intrauterine demise of the fetus occurred intraoperative and the patient underwent surgical uterine evacuation subsequently.

### Patient 2

**Figure 3.**
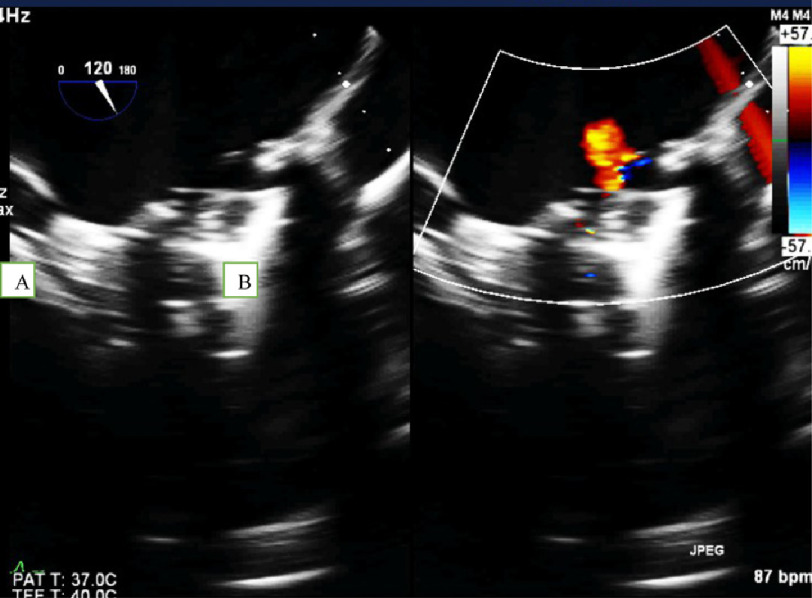
Trans-esophageal echo, mid-esophageal view, angle 120° showing thrombus attached to the mitral prosthesis leaflet (A) and washing jet across obstructed valve (B).

**Figure 4.**
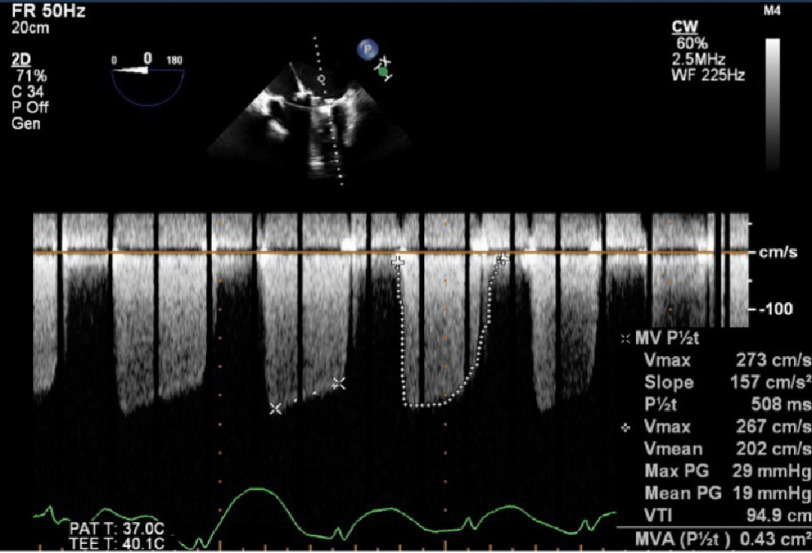
Continuous wave Doppler showing increased gradients across MV (pre-operative).

A 32-year-old female was referred with thrombosed mechanical mitral prosthesis, resulting in cardiogenic shock. She presented at the 13^th^ gestational week of her pregnancy, after shifting from warfarin in the first trimester to low molecular weight heparin, which resulted in thrombosis of the valve (Figure 3 and 4). Emergency surgery was performed and the valve was replaced with a mechanical 29 mm prosthesis. The fetus aborted on seventh day post-operative.

### Patient 3

**Figure 5.**
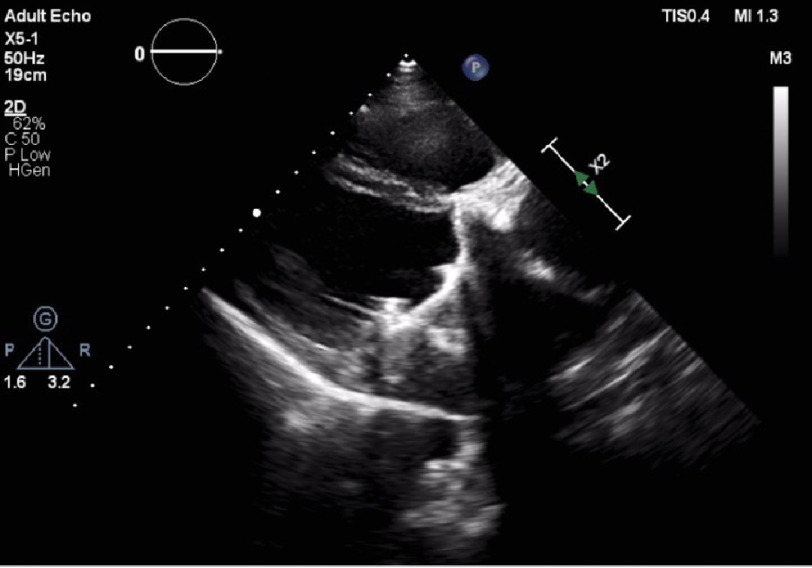
Trans-thoracic echo showing malfunctioning mitral prosthesis.

A 29-year-old female was referred with thrombosed mechanical prosthesis (Figures 4 and 5) one-year post-operative. Patient presented on the 20^th^ gestational week in severe cardiogenic shock, requiring ventilation and vasopressor therapy while on a fixed dose of warfarin (5 mg daily). Fetal death was diagnosed at the time of presentation. Emergency mitral valve replacement was performed, and the patient was discharged to the ICU on high inotropic support. Persistent shock did not resolve and the patient died a few hours post-operative.

### Patient 4

**Figure 6.**
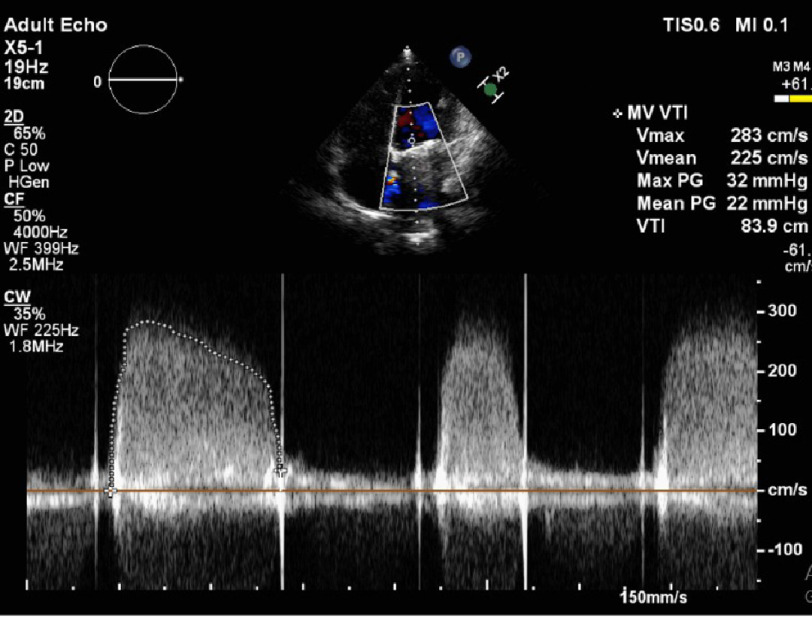
Continuous wave Doppler showing increased gradients across MV (pre-operative).

**Figure 7.**
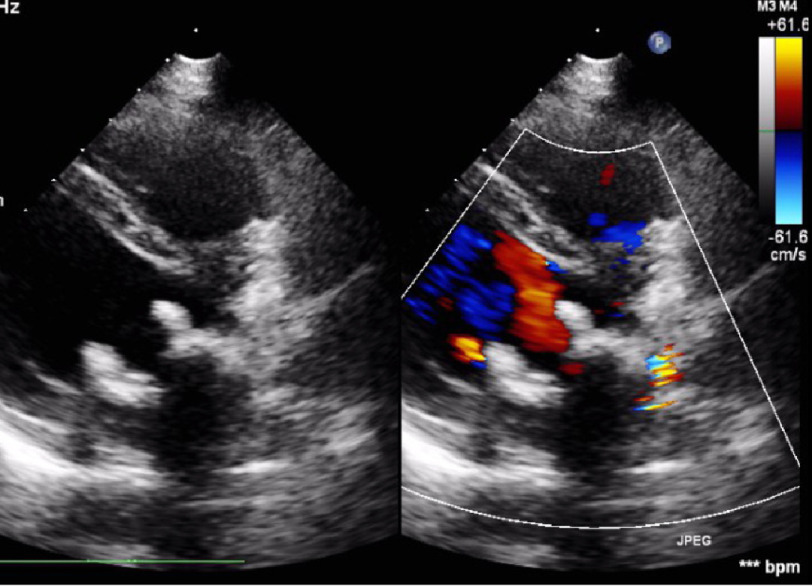
Mitral valve bioprothesis with normal flow across.

A 28-year-old lady presented with thrombosed mechanical prosthesis five years post-operative (Figure 6). There was history of two previous abortions since the cardiac procedure. Patient presented with cardiogenic shock on the 15^th^ gestational week and while compliant to warfarin and within target INR. Emergency valve replacement with biological valve was performed (Figure 7). Patient was discharged from hospital with viable fetus. Unfortunately, during the 28^th^ gestational week, abortion was performed due to an attack of massive vaginal bleeding.

### Maternal morbidity and mortality in relation to mechanical valve prothesis

Due to an increase in successfully corrected congenital heart disease (CHD) and the continuous presence of rheumatic heart disease (RHD), a significant number of women with prosthetic valves are of childbearing age, in whom maternal and fetal morbidity and mortality risks are increased. In non-western countries, rheumatic valvular disease represents up to 89% of all cardiovascular diseases in pregnancy^12–15^.

Pregnant women with prosthetic heart valves, particularly of the mechanical type, are especially at high risk for adverse outcomes^1,16–22^.

The presence of a mechanical valve (WHO risk classification III) is a predictor of maternal cardiovascular and neonatal events^2,6–8^, and anticoagulation regimens carry an increased risk of miscarriage, hemorrhagic complications and teratogenicity^9,10^. Women with prosthetic heart valves are more likely to experience severe maternal morbidity, (more than 9 times higher than matched women without heart disease), with a nonfatal event occurring in 10% of pregnancies, which is at least a 7-fold increase compared with pregnancies in matched controls^22^. Valve thrombosis and hemorrhagic complications occur in 4.7 % and 23.1%^3^, respectively. Maternal mortality in the presence of mechanical heart valves is up to 4%^23^.

The chances of an event-free pregnancy with a live birth in the presence of a mechanical prothesis is only 58%^3^ and favorable outcomes for mother and baby are only seen in 28% of cases^11^.

Maternal adverse outcomes in the presence of a prosthetic heart valve include thromboembolic events, valve thrombosis, cardiovascular compromise, arrythmia, infective endocarditis, obstetric hemorrhage, and maternal mortality^9,10,21^ which is usually related to valve thrombosis^24^.

The fetus is at risk for small gestational age, low birth weight, congenital malformation with the use of anticoagulants, pre-term birth, miscarriage and perinatal mortality including neonatal mortality and stillbirth^21,24–28^.

Mechanical valve thrombosis is a life-threatening situation, and in pregnancy there are no clear treatment options or management in order to lower the risk both for the mother as well as the fetus.

### Prosthetic heart valves

Due to RHD and congenital heart disease a significant number of patients with valvular heart disease, requiring prosthetic valves, are of childbearing age. The diseased native valve can be replaced by a mechanical or bioprosthetic valve, both carrying significant risks and complications. Therefore, care for women with mechanical heart valves in pregnancy poses one of the greatest clinical challenges^3,29^.

Mechanical valve prosthesis is both a cure for a serious disorder as well as a disease itself as it requires lifelong anticoagulation with strict monitoring to protect against thrombo-embolic events. Although, even with the right care, the annual risk for these events remains at around 1%^29^, not to mention the associated risks of bleeding. Importantly, thrombotic risk increases during pregnancy due to the associated pro-thrombotic state, raising the annual risk to 4%^8^.

Mechanical valves show a higher complication rate for both maternal and fetal events compared to bio-protheses^3,30^. The risk decreases from 42% to 22% in the presence of a bioprosthetic valve instead of a mechanical valve^31^. Uncomplicated pregnancies are often seen in patients with well-functioning bioprosthetic heart valves and the absence of other cardiac risk factors due to the fact that they are less thrombogenic than mechanical valves^9,10^. However, the main issue with bioprosthetic valves is their finite lifespan and their risk of structural valvular deterioration (SVD) which can require reoperation in about 50% of women of childbearing age within 10 years of the initial operation^26^. Importantly, pregnancy might also accelerate SVD and reoperation might be required during pregnancy and in the early postpartum period^9,10,26,32^.

Importantly, all patients with prosthetic heart valves (bioprosthetic or mechanical) are at risk of endocarditis^33,34^.

Apart from maternal adverse events and fetal demise in women with cardiac disease, the need for cardiac interventions post-partum was shown to be increased. This can be in the form of ICD and PPM implantation, valve replacement due to the hemodynamic effects of pregnancy on certain valvular lesions, up to LVAD implantation and heart transplantation, which urges the need for continued cardiovascular care in the postpartum period and beyond^35^.

### Anticoagulation

Causes for pregnancy related adverse events in women with prosthetic heart valves are partly attributed to the increased cardiac output during pregnancy and its effect on valve function, alongside the state of increased coagulability leading to the added risk of mechanical valve thrombosis^11,16,17,20,36^. The elevation in circulating pro-coagulant factors and maternal hormones lead to a decrease in prothrombin time, activated partial thromboplastin time, thrombin time and international normalized ratio (INR)^37,38^. Therefore, adequate anticoagulation is more difficult to achieve during pregnancy, increasing the risk of thromboembolic events and mechanical valve thrombosis.

It is also important to take into account the devastating effects of anticoagulation during pregnancy as warfarin is teratogenic and heparin is less effective in preventing thrombotic events compared to warfarin. The risk of thromboembolic events during pregnancy in patients treated with heparin is approximately 10%^9,39^, compared with the 3.9% risk with warfarin use throughout pregnancy^24,40^. Furthermore, the use of unfractionated heparin during pregnancy can be problematic, with an attenuated response of activated partial thromboplastin time (aPTT) and failure to achieve appropriate aPTT levels, variable sensitivities of aPTT reagents, and wide peaks or troughs with the use of subcutaneous unfractionated heparin^39^. The use of warfarin between 6 and 12 weeks’ gestational age results in a 6% to 10% risk of embryopathy and fetal malformations^41,42^; increases the risk for maternal hemorrhage and fetal hemorrhage as it crosses the placenta and shows higher rates of fetal loss^2,24^. Furthermore, the benefits of lowering the daily doses to less than 5 mg/day to decrease risks of embryopathy^29^ remains unconfirmed^1^. Currently, there is no indication for the use of novel oral anticoagulants (NOACs) for mechanical heart valves as the data presented suggests increased bleeding as well as thrombotic events^43^ and there is no data on their risk during pregnancy.

Pregnant patients who suffer a thrombotic or bleeding event while on appropriate anticoagulation therapy are a dilemma as there are no clear options for treatment and surgical interventions are not recommended unless as a last resort. Therefore, the use of thrombolytic drugs such as streptokinase is recommended (for up to 72 hours) in order to solve thrombosis during pregnancy. However, there is limited data available on the success rates of this method and surgery remains generally inevitable if thrombolysis fails. On the other hand, bleeding has no other treatment except discontinuation of the anticoagulant drug or shifting to a less effective agent and accept the risk of thrombosis.

In conclusion, there are no clear guidelines or reliable data from randomized trials for the management of anticoagulation or even its complications during pregnancy. Also, there is no data on how long serum levels for coagulation factors remain increased, which might be a risk for prosthetic valve thrombosis in the period after childbirth. It is also unclear when to restart anticoagulation regimen after childbirth, keeping in mind the risk of bleeding and gynecology complications and valve thrombosis. All of this raises the question whether we should encourage the use of biological valves or the liberal use of mitral valve repair in young females, accepting the risk of re-operation.

### Alternative techniques

Bioprosthetic valves have shown less maternal and fetal risks. However, in order for bioprosthetic valves to be considered for young patients, the morbidity and mortality associated with mechanical valves would need to outweigh the accumulated mortality and inconvenience of re-interventions for bioprosthetic valves. Several Western studies did conclude that there was no difference in long-term mortality between mechanical and tissue valves^44–51^ however, the benefits might be much more prominent in LMIC’s where availability of anticoagulation and medical services might have an impact on clinical outcomes.

In experienced hands, mitral valve repair has shown favorable outcomes, especially in rheumatic heart disease, which is the main reason for valve pathology in the young, and should be considered as the first option for young females.

Hopefully, in the near future, tissue engineered valves (TEHV) may overcome deficiencies of currently available heart valve substitutes. There are approaches being contemplated to generate a living, functional, and durable heart valve structure which could be a potential solution to the shortcomings of the existing mechanical and bioprosthetic valves^52,53^.

### Contraception in the presence of a mechanical heart valve

Pregnancy in women with heart disease is a risky event. Therefore, the choice of contraception requires consideration of pregnancy risk, available contraception options as well as their risks and benefits, failure rates, understanding the consequences of unplanned pregnancy, and the patient’s preferences.

Due to the potential for thromboembolic complications and valve thrombosis, combined hormonal contraceptives in the form of pills, transdermal patches or vaginal rings are not recommended in women with mechanical heart valves^54,55^. In women at prohibitively high risk for pregnancy, permanent forms of contraception can be considered.

Importantly, there is scarce data on contraception for women with heart disease in general and especially those with mechanical prostheses. As a result, physicians may be too cautious and deny contraception, while not being aware of the range of effectivity and safety of various contraceptive methods, leading to the use of less appropriate strategies resulting in unexpected pregnancies and complications of the underlying cardiac disease^56^.

### Possible solutions and future directions

Avoidable deaths from pregnancy related causes, especially due to thrombosis of mechanical heart valves, occur daily on a global scale. Importantly, heart disease is expected to become even a greater contributor to maternal mortality worldwide due to improved survival of women with congenital heart disease and RHD. This results in a significant number of women with prosthetic valves being of childbearing age, in whom maternal and fetal morbidity and mortality risks are increased, due to pregnancy related pro-thrombotic state as well as the dilemma of anticoagulation for mechanical prosthetic valves. Conventional anticoagulation results in significant maternal and fetal morbidity and mortality while heparin is less effective in preventing thrombotic events compared to warfarin.

In the case of thrombosis of the prosthetic valve, the options are limited to streptokinase and surgery in case thrombolysis fails. The maternal and fetal risks of peripartum cardiac surgery cannot be ignored. Our patient series illustrates the dilemma and the risks and outcomes of such procedures. In case of bleeding due to anticoagulation the only treatment option is discontinuation of the anticoagulant drug or shifting to a less effective agent and accept the risk of thrombosis.

The presence of a mechanical heart valve during pregnancy poses therefore one of the greatest clinical challenges for which there are no clear guidelines or reliable clinical data to lean on. Insights on management of women with cardiac disease in the presence of prosthetic valves and the use of anticoagulation are urgently needed.

Bio-protheses show more favorable results regarding pregnancies with less complications, which raises a question whether we should encourage the use of biological valves in young females. However, their finite lifespan and risk of structural valvular deterioration (SVD) leading to reoperation is a concern. Clinical data should analyze the benefits of biological valves during pregnancy as these benefits might be much more prominent in LMIC’s where availability of anticoagulation and medical care is limited.

Mitral valve repair might be a good alternative as it has shown favorable outcomes and should be considered as the first option in young females, accepting the risk of re-operation.

Mothers with heart disease need special care before, during and after pregnancy. Management of women with prosthetic heart valves is required throughout pregnancy and after childbirth in a specialized program for high-risk patients by a multidisciplinary team consisting of an obstetrician, family physician and cardiologist. This multidisciplinary approach should address counselling, medical management, guidance for anticoagulation during pregnancy, protection of the fetus, and importantly, family planning in the future.

This shows the need for specialized maternity clinics for women with prosthetic heart valves in order to improve the quality of care and improve outcomes especially in developing countries where medical care is scarce^59^ and prevention and management will have a huge impact on the overall outcomes.

## Conclusions

This small series of patients serves to illustrate the tip of the iceberg in the dilemma of the combination of mechanical prothesis and pregnancy, which justifies the subtitle of this article “An ongoing “saga” in need of comprehensive solutions.

Thrombosis of mechanical valve prosthesis during pregnancy is still a major problem that carries significant risk of morbidity and mortality. The decision of anticoagulation regimen in pregnancy has to weigh risks of thrombotic events and bleeding versus fetal and maternal safety. The presence of a mechanical heart valve during pregnancy poses therefore one of the greatest clinical challenges for which there are no clear guidelines or reliable clinical data. Insights on management of pregnant women with the combination of prosthetic valves and anticoagulation are urgently needed.

Valve repair, or the use of bio-protheses, show favorable results regarding pregnancies - which raises the question whether we should encourage these alternatives in young females, accepting the risk of re-operation. The maternal benefits for both options should be studied in depth. Contraception, risks of pregnancy, and family planning should be discussed with all patients.

Mothers with heart disease need special care before, during and after pregnancy. Multidisciplinary follow-up in dedicated clinics might lead to a significant reduction in peripartum complications and post-partum mortality. Implementation of these clinics should be encouraged in order to prevent cardiovascular events during pregnancy and beyond.
